# Management of Adenoid Cystic Carcinoma of the Breast: A Single-Institution Study

**DOI:** 10.3389/fonc.2021.621012

**Published:** 2021-03-15

**Authors:** Wenxiang Zhang, Yi Fang, Zhihui Zhang, Jing Wang

**Affiliations:** ^1^ Department of Breast Surgical Oncology, National Cancer Center/National Clinical Research Center for Cancer/Cancer Hospital, Chinese Academy of Medical Sciences and Peking Union Medical College, Beijing, China; ^2^ Cytology Section Department of Pathology, National Cancer Centre/National Clinical Research Center for Cancer/Cancer Hospital, Chinese Academy of Medical Sciences and Peking Union Medical College, Beijing, China

**Keywords:** adenoid cystic carcinoma, breast cancer, clinicopathological features, treatment, prognosis

## Abstract

**Objective:**

The purpose of our study was to analyze the clinicopathologic features and surgical and oncological outcomes of adenoid cystic carcinoma (ACC) of the breast and to provide the basis for a clinical therapeutic schedule.

**Methods:**

A total of 14 patients with primary breast adenoid cystic carcinoma treated at Cancer Hospital of the Chinese Academy of Medical Sciences from January 2000 to December 2017 were included. Data on clinical presentation, treatment strategy, and outcome, as well as the pathological features of ACC, were reviewed and analyzed.

**Results:**

Fourteen patients were diagnosed with ACC of the breast, out of 23205 total patients treated for breast cancer (0.06%). All but three patients were postmenopausal, with a median age at diagnosis of 60.5 years (range, 39–73 years). The most common clinical presentation was a palpable mass (85.7%), and the imaging characteristics of all patients on color Doppler ultrasound and mammography were nonspecific. Six patients (42.9%) were suspected of having ACC by fine-needle aspiration cytology (FNAC) and were confirmed by postoperative histology and immunohistochemistry. All 14 patients underwent surgery, and no patient had a positive lymph node status. Median tumor size was 1.75 cm (range, 1–3 cm). Eight/14 (57.1%) patients were hormone receptor negative (HR−) and HER-2/neu (−) (HER2−). The remaining patients were hormone receptor positive (HR+). There was no significant difference in clinicopathological characteristics between the HR+ group and the HR- group (P>0.05). The mean follow-up period was 57 months. Local recurrence occurred in 14.3% of patients, 1.7% of patients had distant metastasis, all patients with local recurrence or distant metastasis were in the HR (-) group, and all patients were alive at the last follow-up.

**Conclusion:**

ACC of the breast cannot be simply summarized as triple-negative breast cancer because it also includes a small number of hormone receptor-positive breast cancers. Establishing a preoperative diagnosis is difficult on the basis of clinical imaging examination, FNAC may be useful tool in the diagnosis. the final diagnosis can only be assessed based on the results of the histopathological and immunohistochemical examination. Breast-conserving surgery may be an alternative treatment strategy, and axillary lymph node dissection or sentinel node biopsy may not be necessary in some cases.

## Introduction

Adenoid cystic carcinoma (ACC) of the breast is a rare special histological type of breast cancer, accounting for approximately 0.1% of all breast tumors (1–3). Most cases are in females, and the median age of onset is between 50 and 60 years old (4, 5). However, occasional cases have been reported in male patients (6, 7). The typical clinical feature is a single mass of the breast, and multiple nodules are rare. Most ACCs are located under the areola or in the upper outer quadrants (4, 8, 9). ACC of the breast has no characteristic imaging findings. Ultrasound features are those of a hypoechoic solid or heterogeneous mass. On mammography, the case may present as a lobulated mass with sharp or unsharp margins (8). Nevertheless, these clinical and radiographic features may be similar to any breast cancer, thus making their precise diagnosis difficult for radiologists (2).

Histologically, ACC of the breast typically consists of a dual-cell population of luminal and myoepithelial-basal cells, which are generally negative for estrogen receptor (ER), progesterone receptor (PR), and human epidermal growth factor receptor 2 (HER2) (2, 10). However, the prognosis of ACC of the breast is usually better than that of other triple-negative breast cancers (4, 11). As prognosis is good, accurate preoperative diagnosis is important in the determination of suitable treatment. In addition, some studies have also reported some HR-positive ACC cases (5). The significance of a positive hormone receptor status is not known. Compared with ACC with negative HR expression, the clinical characteristics and prognosis of this type of ACC are also unknown.

One major obstacle when optimizing the therapeutic management of ACC is the rarity of the tumor. As a result, no consensus exists on the optimal therapy. This study presents the clinical manifestations, imaging characteristics, pathological findings, and surgical and oncological outcomes of breast ACC in patients seen at a single institution. At the same time, we assessed the utility of FNAC in the diagnosis of ACC of the breast. We also describe the clinicopathological features of HR+ ACC and compare them with those of HR- breast ACC. To the best of our knowledge, this study is one of the few that focuses on ACC with HR+ status.

## Materials and Methods

From January 2000 to December 2017, a retrospective analysis revealed that 23,205 patients were diagnosed with breast cancer and surgically treated at XXX. Among these patients, a total of 14 patients were pathologically diagnosed with adenoid cystic carcinoma of the breast. We summarized the clinical and pathological data (age, sex, tumor size, tumor location, immunohistochemical features), management (surgical, chemotherapy, endocrine therapy, and radiation), estrogen and progesterone receptor status, and prognostic information of 14 patients with ACC and conducted statistical analysis.

In this study, ER status was determined by immunohistochemistry and defined as positive with a cut-off of 1%. HER2 status was defined as positive if scored as 3+ on immunohistochemistry or if fluorescence *in situ* hybridization demonstrated gene amplification. Three histological grades were determined according to WHO classification: Grade I, tumors with tubular and cribriform areas, but without solid components; grade II, cribriform tumors that were either pure or mixed with <30% solid areas; and grade III, tumors with >30% solid patterns.

Descriptive statistics are reported as frequencies and percentages. Differences between HR+ ACC and HR- ACC with regard to clinicopathological features were evaluated using the chi-square test, and P < 0.05 was considered statistically significant in all the analyses. All statistical analyses were performed using SPSS 26.0 software (IBM Corporation, Armonk, NY, USA). Overall survival was defined as the time from the first day of therapy to the date of death; if the patient was alive at the end of the follow-up period or was lost to follow-up, OS was censored on the last date the patient was known to be alive. Disease-free survival (DFS) was also calculated from the date of first diagnostic biopsy, with first recurrences, local or distant, being scored as an event, and with censoring of other patients at the time of last follow-up or death. DFS curves were drawn using Kaplan-Meier estimates and the curves were compared by the log-rank test. The study received approval from the Institutional Review Board of our institution, and patient consent was obtained.

## Results

### Clinical Features

The study identified 14 out of 23,205 patients (incidence, 0.06%) who underwent breast cancer surgery from January 2000 to December 2017. **Table 1** lists clinical characteristics. All patients were female. Among the 14 patients, only three were premenopausal, and the remaining were postmenopausal. The median age of the diagnosis was 60.5 years, with a range of 39–73 years. There was no family history of breast cancer in any patients. The first symptom of all 14 patients was breast masses, of which one was accompanied by nipple discharge and one was accompanied by pain. The tumors were located in the right breast in nine cases and the left breast in five cases. The tumors located in the quadrant were eight cases in the upper outer quadrant, one case in the lower inner quadrant, two cases in the lower outer quadrant, and three cases in the central quadrants. Two tumors were found through routine screening, and 12 patients had found the tumor by themselves.

**Table 1:** Baseline characteristics.

Characteristic	n = 14
**Incidence (% of all breast cancer)**	0.06%
**Mean age at diagnosis (range)**	60.5 (39–73)
**Female, n (%)**	14 (100)
**Menopausal status, n (%)**	
Premenopausal	3 (21.4)
Postmenopausal	11 (78.6)
**Laterality, n (%)**	
Left	5 (35.7)
Right	9 (64.3)
**Tumor distribution, n (%)**	
Upper outer quadrant	8 (57.1)
Upper inner quadrant	0 (0)
Lower inner quadrant	1 (7.1)
Lower outer quadrant.	2 (14.3)
Central quadrants	3 (21.4)
**Symptoms, n (%)**	
Palpable mass	10 (71.4)
Palpable mass with pain	1 (7.1)
Palpable mass with nipple Discharge	1 (7.1)

### Preoperative Examination

**Figure 1:**
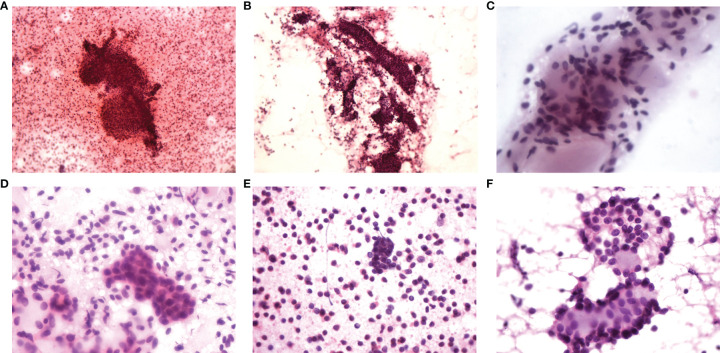
FNAC findings of six cases. **(A)** Tubular and sieve-shaped epithelial cells; **(B)** Sieve-shaped epithelial cells; **(C)** Sieve-shaped epithelial cells; **(D)** Sieve-shaped epithelial cells; **(E)** Pieces of epithelial cells and mucoid globules; **(F)** Sieve-shaped epithelial cells.

Thirteen of the 14 patients underwent bilateral breast ultrasound. The mammogram was performed in five patients. Magnetic resonance imaging (MRI) of the breasts was performed in three patients. The imaging findings of ACC are summarized in **Table 2**. Tumors ranged in size from 0.6 to 2.81 cm with a median size of 1.6 cm on breast ultrasound. The sonographic characteristics of the ACC of the breast were all hypoechoic solids. Of the 13 patients who underwent breast ultrasound, 10 presented with an irregular mass. Mammograms were available for five of the 14 patients, and masses were identified in all cases. The tumor presented as an irregular or lobular mass with spiculated or indistinct margins. Small calcifications were found in one case. Only three patients underwent MRI, and all patients presented with an irregular mass. Compared with normal breast tissue, all lesions appeared homogeneously isointense on T1WI. After the injections of contrast agent, the enhancement of all masses was rapid and heterogeneous (patient 5) or rapid and homogenous (patient 10 and patient 11). On T2-weighted imaging (T2WI), one patient (with a 2.06-cm mass) had extensive high T2WI signals and hypointense internal septations, which demonstrated plateau kinetics. In the other two cases with smaller lesions (with a 1.0-cm mass), MRI showed that these lesions were isointense on T2 imaging; one mass demonstrated washout kinetics, and the other demonstrated plateau kinetics. Six patients underwent fine-needle aspiration cytology (FNAC), four underwent excisional biopsy, and four underwent core needle biopsy (CNB). Among the 6 cases of fine-needle aspiration, sieve-shaped epithelial cells were observed in five patients, and pieces of epithelial cells and mucoid globules were observed in one patient (**Figure 1**). In all cases, ACC was suspected. In postoperative histological sections, these cytological findings were compatible with the histological findings and were diagnosed as breast ACC. Only one patient was diagnosed with invasive breast cancer through the CBN, which had a biopsy in another hospital and did not undergo the immunohistochemical examination. The rest were diagnosed with ACC of the breast; microscopically, the tumor cells consisted of epithelial and myoepithelial cell types arranged into tubular or cribriform architecture.

**Table 2:** Preoperative examination findings of patients with ACC of the breast.

Patient	US findings				Mammography findings		MRI			Preoperative pathological diagnosis		
	Size (cm)	Boundary	Internal blood vessel	Shape	Size (cm)	Features	T2-weighted signal intensity	InternalEnhancement	Kinetics	Diagnostic method	Features	Conclusion
1	1.2	Unclear	Yes	Irregular	1.7	Irregular mass with indistinct margins; No calcifications	None	None	None	FNAC	Tubular and sieve-shaped epithelial cells	Suspected tubular cancer or adenoid cystic carcinoma
2	1.8	Unclear	Yes	Irregular	Not done	Not done	None	None	None	FNAC	Sieve-shaped epithelial cells	Suspicious for ACC
3	1.9	Unclear	Yes	NR	2.3	Irregular mass; No calcifications	None	None	None	FNAC	Sieve-shaped epithelial cells	Suspicious for ACC
4	0.9	Clear	Yes	Regular	Not done	Not done	None	None	None	FNAC	Pieces of Epithelial cells and mucoid globules	Suspicious for ACC
5	2.06	Unclear	Yes	Irregular	Not done	Not done	Hypointense	Rapid and heterogeneous	Plateau	CNB	None	Invasive carcinoma
6	1.8	Unclear	No	Irregular	1.8	Irregular mass with indistinct margins, No calcifications	None	None	None	Excisional biopsy	Tumor cells contain mesh-like cyst-like cavities	ACC
7	1.7	Unclear	Yes	Irregular	Not done	Not done	None	None	None	CNB	Tumor cells appear as small tubes	ACC
8	1.1	Unclear	Yes	Irregular	Not done	Not done	None	None	None	CNB	None	Suspicious for ACC
9	1.6	Unclear	Yes	Irregular	2.1	High-density nodule shadow; Irregular shape; No calcification	None	None	None	FNAC	Sieve-shaped epithelial cells	Suspicious for ACC
10	1	Unclear	No	Irregular	Not done	Not done	Isointensity	Rapid and homogeneous	Washout	Excisional biopsy	Tumor cells contain mesh-like cyst-like cavities	ACC
11	1	Unclear	Yes	Irregular	Not found	Not done	Isointensity	Rapid and homogeneous	Plateau	CNB	Tumor cells contain mesh-like cyst-like cavities	ACC
12	1.6	Unclear	NR	Irregular	Not done	Not done	None	None	None	Excisional biopsy	Tumor cells appear as a cord	ACC
13	Not done	Not done	Not done	Not done	Not done	Not done	None	None	None	Excisional biopsy	Tumor cells contain mesh-like cyst-like cavities	ACC
14	2.81	unclear	Yes	irregular	3.2	Lobular mass with spiculated margins; Small calcifications	None	None	None	FNAC	Sieve-shaped epithelial cells	ACC

FNAC, fine-needle aspiration cytology; CNB, core needle biopsy; ACC, adenoid cystic carcinoma.

### Pathological Findings

All cases were diagnosed by two senior pathologists. Of the 14 patients, 13 had pure ACC, and one had ACC with ductal carcinoma *in situ* (DCIS). On histopathology, most tumors in our study formed by myoepithelial cells and glandular epithelial cells in a biphasic pattern which revealed a tubular subtype in three cases, a cribriform in 10 cases and a solid subtype in one case. Grade 1 was observed in nine of 14 cases (64.3%), grade 2 in three of 14 cases (21.4%) and grade 3 in one of 14 (7.2%). The average maximum diameter of the tumor was 1.75 cm (1.0-3.0 cm). None of the cases had vascular tumor thrombus, and perineural invasion was present in three cases. The median number of lymph nodes examined was 5 (range, 1–29), and no patient had a positive lymph node status. In total, all patients had early-stage breast cancer (stages IA and IIA). Eight patients’ ACCs were TNBC, while six patients were HR+/HER2−. Of the six ACCs that were HR+/HER2−, four cases were ER+ with PR+, two cases were ER- with PR+. The Ki67 proliferation index was less than 14% in 71.4% of the cases analyzed for Ki67 (**Table 3**). Meanwhile, we compared the HR+ACC group with the HR+ACC group. A significant association between the two groups was not found in age, menopausal status, tumor size, clinical stage of surgical methods, KI67, progesterone receptor status, history of chemotherapy or radiotherapy (P>0.05). all information can be found in **Table 4**. As for immunohistological features, all patients were all CD117 (c-Kit) positive. immunohistochemical staining indicated CK5/6, p63 positive results in nine cases, 34-Beta-12 positive in two cases, S-100 in four cases, Calponin positive in four cases. The summary of all the findings is shown in **Supplementary Table 1**.

**Table 3:** Pathologic characteristics of 14 patients diagnosed with ACC of the breast.

Patient	Tumor size (cm)	Histological grade	Tumor Thrombus	Perineural invasion	No. of resected LNs	Lymph node metastasis	TNM stage	Clinical stage	ER and PR status	HER-2
1	2.5	Unknown	No	No	29	pN0	T2N0M0	IIa	ER- PR-	Negative
2	2.1	I	No	No	28	pN0	T2N0M0	IIa	ER+ PR+	Negative
3	1.7	I	No	No	4	pN0	T1cN0M0	Ia	ER- PR-	Negative
4	1.3	I	No	No	17	pN0	T1cN0M0	Ia	ER+ PR+	Negative
5	2.2	I	No	Yes	7	pN0	T2N0M0	IIa	ER+ PR-	Negative
6	1.8	I	No	No	4	pN0	T1cN0M0	Ia	ER- PR-	Negative
7	2.2	II	No	No	3	pN0	T2N0M0	IIa	ER- PR-	Negative
8	1.1	I	No	No	3	pN0	T1cN0M0	Ia	ER+ PR-	Negative
9	1.7	I	No	Yes	4	pN0	T1cN0M0	Ia	ER+ PR-	Negative
10	1	III	No	No	9	pN0	T1bN0M0	Ia	ER- PR-	Negative
11	1.5	II	No	No	6	pN0	T1cN0M0	Ia	ER+ PR-	Negative
12	1.6	I	No	No	5	pN0	T1cN0M0	Ia	ER- PR-	Negative
13	2	II	No	No	None	cN0	T1cN0M0	Ia	ER- PR-	Negative
14	3	I	No	Yes	1	pN0	T2N0M0	IIa	ER- PR-	Negative

ER, estrogen receptor; PR, progesterone receptor; HER2, human epidermal growth factor receptor 2.

**Table 4:** Comparison of clinicopathological features of HR+ group and HR- group.

Characteristics	HR-group(n=8,%)	HR+ group(n=6,%)	Total (n=14, %)	P value
**Age (years)**				
<60.5	5 (35.7)	4 (28.6)	9 (64.3)	0.872
≥60.5	3 (21.4)	2 (14.3)	5 (35.7)	
**Year diagnosed**				
2000–2015	5 (35.7)	2 (14.3)	7 (50.0)	0.28
2015–2017	3 (21.4)	4 (28.6)	7 (50.0)	
**Menstrual status**				
Premenopausal	1 (7.1)	2 (14.3)	3 (21.4)	0.347
Postmenopausal	7 (50.0)	4 (28.6)	11 (78.6)	
**Tumor size (cm)**				
≤2 cm	5 (35.7)	4 (28.6)	9 (64.3)	0.872
2–5 cm	3 (21.4)	2 (14.3)	5 (35.7)	
>5 cm	0 (0)	0 (0)	0 (0)	
**Axillary staging**				
N_0_	8 (57.1)	6 (42.9)	14 (100)	N/A
**Perineural invasion**				
Yes	1 (7.1)	2 (14.3)	3 (21.4)	0.347
N0	7 (50.0)	4 (28.6)	11 (78.6)	
**Ki 67**				
≤14%	7 (50.0)	3 (21.4)	10 (71.4)	0.124
>14%	1 (7.1)	3 (21.4)	4 (28.6)	
**Histological grade**				0.531
I	4 (28.6)	5 (35.7)	9 (64.3)	
II	2 (14.3)	1 (7.1)	3 (21.4)	
III	1 (7.1)	0 (0)	1 (7.1)	
Unknown	1 (7.1)	0 (0)	1 (7.1)	
**Surgical procedure**				
BCS or WLE	2 (14.3)	2 (14.3)	4 (28.6)	0.733
MT	6 (42.9)	4 (28.6)	10(71.4)	
**Clinical stage**				
I	5 (35.7)	4 (28.6)	9 (64.3)	0.266
II	3 (21.4)	2(14.3)	5 (35.7)	
**Adjuvant radiotherapy**				
Yes	2 (14.3)	2 (14.3)	4 (28.6)	0.733
No	6 (42.9)	4 (28.6)	10 (71.4)	
**Adjuvant chemotherapy**				
Yes	1 (7.1)	0 (0.0)	1 (7.1)	0.369
No	7 (50.0)	6 (42.9)	13 (92.9)	
**Local recurrence**				
Yes	2 (14.3)	0	2 (14.3)	0.186
No	6 (42.9)	6 (42.9)	12 (85.7)	
**Distant recurrence**				
Yes	1 (7.1)	0 (0.0)	1 (7.1)	0.369
No	7 (50.0)	6 (42.9)	13 (92.9)	

HR, hormone receptor; BCS, breast-conserving surgery; ALND, axillary lymph node dissection; SLNB, sentinel lymph node biopsy; MT, Mastectomy; WLE, wide local excision; N/A, not applicable.

### Treatment and Prognosis

All patients underwent surgery: four patients underwent radical mastectomy with axillary lymph node dissection (ALND), six patients underwent simple mastectomy with sentinel lymph node biopsy (SLNB), three patients underwent breast-conserving surgery (BCS) with SLNB, and only one patient underwent wide local excision (WLE). Five of six patients with hormone receptor-positive tumors received hormonal therapy. Chemotherapy was performed in two patients. Of the four patients who underwent locoregional radiotherapy, all were in the BCS or WLE group (**Table 5**).

**Table 5:** Treatment and prognosis of patients with ACC of the breast.

Patient	Surgery	CT	RT	HT	Local Recurrence	DistantMetastasis	DFS (mo)	OS (mo)	Prognosis
1	MT+ALND	Yes	No	No	No	No	115	115	Alive
2	MT+ALND	No	No	Yes	No	No	95	95	Alive
3	MT+SLNB	No	No	No	No	No	95	95	Alive
4	MT+ALND	No	No	Yes	No	No	83	83	Alive
5	MT+ALND	No	No	Yes	No	No	59	59	Alive
6	MT+SLNB	No	No	No	No	No	64	64	Alive
7	MT+SLNB	No	No	No	No	No	50	50	Alive
8	BCS+SLNB	No	Yes	Yes	No	No	34	34	Alive
9	MT+SLNB	No	No	No	No	No	48	48	Alive
10	MT+SLNB	Yes	No	No	Yes	Yes (lung)	11	40	Alive
11	BCS+SLNB	No	Yes	Yes	No	No	32	32	Alive
12	BCS+SLNB	No	Yes	No	No	No	25	25	Alive
13	WLE	No	Yes	No	Yes	Lapses	6	–	–
14	MT+SLNB	No	No	No	No	No	148	148	Alive

CT, chemotherapy, RT, radiotherapy; HT, hormone therapy; DFS: disease-free survival; OS, overall survival; BCS, breast-conserving surgery; ALND, axillary lymph node dissection; SLND, sentinel lymph node biopsy; MT, Mastectomy; WLE, wide local excision.

**Figure 2:**
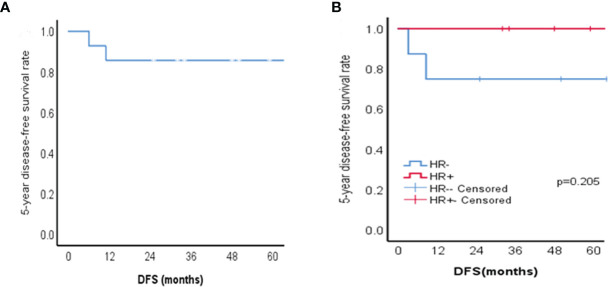
Kaplan-Meier survival curves of patients with ACC of the breast. **(A)** 5-year disease-free survival (DFS) for 14 patients with ACC of the breast; **(B)** The 5-year disease-free survival of HR + group and HR - group in patients with ACC of the breast (*p*=0.205).

The median follow-up was 59 months (range 25–148 months). None of the patients had recurrence within the axilla. One patient (case 10) who experienced recurrence 11 months after the operation presented local recurrence again over the following 5 months and underwent two resections of chest wall recurrences while discovering lung metastases simultaneously in the second relapse. During disease progression, she was successively treated with doxorubicin/cyclophosphamide, docetaxel/capecitabine/nituzumab, and gemcitabine/carboplatin. Fortunately, during our follow-up, this patient was still alive. The other patient had recurrence of the breast at 6 months after the operation, but after the second operation, she was lost to follow-up. Lapses occurred in the HR-ACC group. In all patients, the 5-year DFS was 85.7%, and **Figure 2** shows the Kaplan-Meier survival curve.

## Discussion

Adenoid cystic carcinoma is a malignant tumor that occurs in the exocrine glands and in body parts with glands, including the salivary glands, lungs, prostate, and breasts (10). For breast ACC, a previous study using SEER data (1977-2006) showed that the age-adjusted incidence ratio (AAIR) of breast ACC is approximately 0.92 per 1 million person-years (11). In view of the rarity of this disease, there is a lack of understanding of the occurrence and development of the disease, and no consensus has been reached regarding clinical diagnosis and treatment. The current study reviewed 14 patients with breast ACC in a single institute during a 17-year period and analyzed the clinical presentations, imaging features, pathological characteristics and treatment outcomes. Demographically, the incidence of ACC of the breast was approximately 0.06% in this study. All patients were female, 85.7% of patients were postmenopausal, and the median age of diagnosis was 60.5 years. These characteristics are similar to those reported in previous studies (11, 12).

The clinical presentation of breast ACC is nonspecific, making the diagnostic process challenging when only clinical features or imaging findings are available. The most common clinical presentation is a palpable mass (12–15). Other rare manifestations include breast pain, nipple discharge, and nipple retraction (14). Both sides can be affected, and most of them are located in the submastoid region or in the upper part of the outer quadrant (8). In this study, palpable masses accounted for 87.5%, most of which were located in the upper part of the outer quadrant. One case was associated with nipple discharge, and the other was associated with breast pain. Imaging features of breast adenoid cystic carcinoma have been reported, however, Imaging characteristics are nonspecific and vary widely, resulting in relatively scarce information on the value of diagnosis and treatment through imaging features. Mammographically, these tumors may appear as irregular or lobulated mass with indistinct or spiculated margins (16). Sonographically, they appear hypoechoic and heterogeneous mass with unsharp margins (1), which is consistent with our study. In addition, the characteristics of MRI in adenoid cystic carcinoma of the breast are rarely reported and controversial. Tang W et al. (8) evaluated 11 patients**’** imaging examinations and found that a well-defined border, extensive high T2WI signals, and internal septations that demonstrate delayed enhancement in larger lesions, which are some valuable manifestations emerged on MRI, more interestingly, in their study, most patients showed benign lesions (four of nine had oval shapes, and seven of nine had smooth margins) on MRI. This is different from other studies, Glazebrook et al. (1) found that on MRI, the masses appeared as irregular shapes with spiculated margins. Kasagawa T et al. (16) described it as a round, oval, or irregular lesion with rapid enhancement (no wash-out) that extends from the margin to the center over time. In our study, only three patients underwent MRI. the masses showed irregular shapes, unenhanced scans showed that the mass of 2.06 cm demonstrated extensive hyperintensity on T2WI and that the mass of 1 cm demonstrated isointensity on T2WI, this information is similar to some results of Tang W et al. and Glazebrook et al. However, can these imaging features be the unique manifestations of adenoid cystic carcinoma of the breast, especially the imaging features on MRI, more analytical clinical studies are worthwhile in the future.

As one of the important methods for preoperative qualitative diagnosis of breast cancer, FNAC has a similar effect to pathological examination, and its diagnostic value has been generally recognized. However, for breast adenoid cystic carcinoma, only a few case reports or a small number of sample studies describe the application of FNAC in ACC diagnosis (6, 17–22). The diagnostic cytological findings of ACC are rarely described. There are only scattered descriptions, such as the cell smear having a single round circular hyperchromic cell, which exists in loose clusters, or the tumor cells surround the nucleus and sphere without cell homogeneous material (21, 22), so there is no uniform conclusion on the diagnostic value of FNAC in ACC. Quinodoz et al. **(** 18 **)** reported that immunocytochemistry may provide additional information on FNAC, which could be relevant not only for diagnosis but also for treatment and prognosis. Ilkay et al. **(** 22 **)** first reported a case of breast ACC that was diagnosed on the basis of immunohistochemical staining of FNAC cell block material. In our study, 42.9% of patients underwent FNAC before surgery. One of them needed to be differentiated from tubule cancer, and the others were highly suspected of ACC, all of which were confirmed histologically after surgery. Features of ACC in our study included cellular smear (uniform, round hyperchromatic cells) surrounded by epithelial cells with little cytoplasm and small hyperchromatic nuclei, from our experience, with these cytological characteristics, we can highly suspect ACC of the breast, if there is immunocytochemistry, this diagnosis is more convincing. Regrettably, cell block material or immunohistochemical staining was not performed on the tumor at the time, which may be part of the factor in the failure to truly diagnose ACC preoperatively.

Histologically, ACC of the breast has a biphasic pattern that consisted of true laminae and pseudo cystic spaces. True glands are lined by epithelial cells and pseudocysts are lined by myoepithelial cells, which are in various morphological configurations, including tubular, cribriform and solid patterns (23). The vast majority of studies show that the cribriform and solid histological patterns are the dominant patterns while a tubular pattern is less commonly. Previous studies found that cribriform and solid patterns were thought to predict more biological aggressiveness while tubular pattern represented a more differentiated pattern of ACC (24). Sequeiros et al. reported that patients with solid histological type had the worst prognosis, the cribriform type was in the middle, and the tubular type was the best (25). In our study, the main histological subtypes were the cribriform pattern in 10 patients (71.4%), tubular pattern in three patients (21.4%), and solid pattern in the remaining one patient (7.1%). The histological subtype of patients with metastasis and recurrence are solid pattern and cribriform pattern. Furthermore, previously Scattered reports found these tumors are usually ER-negative and PR negative (3, 13, 26). However, Arpino G et al. reported 28 patients and found that ER and PR expression were detected in 46% (13 of 28) and 36% (10 of 28) of ACC cases, respectively (27). In the current series of 14 cases, 28.5% were found to be ER-positive (four of 14) and 14.3% were PR positive (2 of 14). We compared the HR+ACC group with the HR+ACC group and found that there was no significant difference in clinicopathologic characteristics between the two groups, suggesting that perhaps a positive ER and PR status does not significantly affect the prognosis of the tumor. Because ACC of the breast is relatively rare, it is not realistic to develop standard treatment protocols through large-scale randomized clinical trials. Until now, the best treatment for ACC has remained controversial. In some early studies, most scholars recommended mastectomy as the initial treatment because of the low local recurrence rate (28, 29). However, in the context of comprehensive treatment, especially the application of radiotherapy technology, breast-conserving surgery (BCS) has become another choice for breast ACC (27, 30, 31). In the study of Arpino et al., 6 patients (21.4%) were treated by lumpectomy, and five patients received adjuvant RT after lumpectomy. all were no local recurrences (27). Khanfir K et al. (4) studied 61 patients with breast ACC and found that patients who had BCS + RT had significantly improved locoregional function compared to patients who had undergone BCS only (95% vs. 83%, p =0.03). There was no significant difference in the 5-year actuarial local control rate between BCS+RT and mastectomy (p=0.16). Furthermore, a recent study using SEER data (1998-2011) evaluated the benefit of radiotherapy in patients with breast ACC and found that patients treated with lumpectomy + adjuvant RT had better survival rates than patients who underwent lumpectomy only (CSS, p=0.018; OS, p = 0.031) or mastectomy only (CSS, p =0.010; OS, p =0.004) (12). which suggests that BCS and adjuvant RT may be the optimal local treatment procedure in patients with ACC of the breast (12). In our study, 4 patients (28.6%) underwent breast-conserving surgery + local radiotherapy. Compared with the prognosis of patients with mastectomy, the results of this treatment showed similar outcomes. Although the proportion of this treatment mode seems to be relatively low, it is not difficult to find that all four patients were diagnosed and treated after 2015. In addition, with regard to the choice of local surgical treatment for ACC, doctors’ preferences may also be mixed.

ACC of the breast has a very low rate of axillary lymph node metastasis. In a study using the California cancer registry, 5% of patients had lymph node involvement (13). Even in other studies, none of the cases found axillary lymph node metastasis through sentinel lymph node biopsy or axillary lymph node dissection (3, 5). Thus, some studies recommend that axillary surgery might be omitted safely in patients with pure ACC and a clinically negative axilla. In our study, axillary operations were sentinel lymph node surgery in nine cases (64.3%) and axillary lymph node dissection in four cases (28.6%). All patients who completed axillary surgery (n = 13) were negative on the final pathology. This result suggests that our treatment of axillary lymph nodes may be excessive to some extent. In other words, axillary surgery might be omitted in patients in the future, but the choice of patients must be made cautiously. The value of systemic adjuvant chemotherapy for ACC of the breast has not been established. Systemic adjuvant chemotherapy can be considered for breast ACC patients with lymph node metastasis (greater than micrometastasis), tumor size **>**3 cm or high lesion grade. Two of our patients received adjuvant chemotherapy, of which one patient had undergone MT+ ALND followed by systemic adjuvant chemotherapy. Another patient chose adjuvant chemotherapy due to local recurrence of the chest wall. However, we have no way to determine whether such systemic adjuvant chemotherapy can benefit survival due to the lack of a control and a small sample size.

Contrasted with other triple-negative, basal-like breast cancers and the ACC of the salivary gland, ACC of breast have a good prognosis. An increasing body of study has shown that the 5-year disease-free survival rate of breast ACC ranges from 82%-100% (4, 27, 30), and the 5-year overall survival rate was more than 80% (4, 13, 27, 30). Macy M et al. reviewed related breast-ACC studies and found that Crude local recurrence and distant recurrence rates were 9.9% and 5.5%, respectively, However, in their research, the unadjusted local and distant recurrence rate were 22.2% and 11.1%, respectively (32). In our study, the crude local and distant recurrence rates were 14.3% and 7.1%, respectively, at a median follow-up of 57 months. These uneven prognostic data suggest that ACC may not be a completely indolent TNBC subtype, which brings into question the best method of surveillance in these patients. Some studies have found that in the clinical course of ACC, the risk of secondary malignant tumors increases and that recurrence and metastasis will occur in the long term, so long-term follow-up is necessary.

## Conclusion

In conclusion, our results show that the majority of ACCs were triple-negative, but our study also included a small number of hormone receptor-positive breast cancers. Compared with other types of breast cancer, ACC has no specificity in imaging, FNAC may be useful tool in the diagnosis. the final diagnosis can only be assessed based on the results of the histopathological and immunohistochemical examination. In our research, we found that BCS + RT may be the optimal local treatment for ACC. Such results need to be interpreted with caution because this was a single-center retrospective study, and the number of cases was small. In view of these limitations, the relevant conclusions should be further confirmed by larger scale studies.

## Data Availability Statement

The original contributions presented in the study are included in the article/**Supplementary Material**. Further inquiries can be directed to the corresponding authors.

## Ethics Statement

The studies involving human participants were reviewed and approved by The authors are accountable for all aspects of the work in ensuring that questions related to the accuracy or integrity of any part of the work are appropriately investigated and resolved. This study has been approved by ethical review by the ethics committee of Cancer Hospital Chinese Academy of Medical Sciences. All procedures performed in studies involving human participants were in accordance with the ethical standards of the institutional and/or national research committee(s) and with the Helsinki Declaration (as revised in 2013). All subjects gave written informed consent in accordance with the Declaration of Helsinki. The patients/participants provided their written informed consent to participate in this study.

## Author Contributions

YF and JW contributed to the conception and design of the study. WZ wrote the first draft of the manuscript. ZZ performed the pathological investigation. All authors contributed to the article and approved the submitted version.

## Funding

This study was supported by the National Natural Science Foundation of China (No.81872160). The above funders had no further role in the study design; collection, analysis, and interpretation of data; writing of the manuscript; or decision to submit this manuscript for publication.

## Conflict of Interest

The authors declare that the research was conducted in the absence of any commercial or financial relationships that could be construed as a potential conflict of interest.
