# Supplementary material for: Management of Adenoid Cystic Carcinoma of the Breast: A Single-Institution Study
Source: Front Oncol. 2021 Mar 15;11:621012. doi: 10.3389/fonc.2021.621012 (PMC8005703; doi:10.3389/fonc.2021.621012)
Supplement: Supplementary file 1 [file Table_1.docx]

| **Supplementary Table 1**. Immunohistochemistry findings of patients with ACC of the breast | | | | | | | | | | | | | | | | | | |
| --- | --- | --- | --- | --- | --- | --- | --- | --- | --- | --- | --- | --- | --- | --- | --- | --- | --- | --- |
| Patient | ER | PR | HER-2 | Ki-67 | CD117 | CK7 | CK34B E12 | CK5/6 | P63 | Calponin | PAS | S-100 | P53 | E-cadherin | SMA | EGFR | TOP2A |  |
| 1 | Negative | Negative | Negative | 5% | 2+ | None | None | None | None | Negative | None | Negative | None | None | None | None | 1+ |  |
| 2 | Positive | Positive | Negative | 25% | 2+ | 3+ | 3+ | 3+ | 3+ | 1+ | 1+ | None | None | None | None | None | None |  |
| 3 | Negative | Negative | Negative | 10% | 1+ | None | None | Focal (1+) | None | None | None | None | 1+ | None | None | 2+ | 1+ |  |
| 4 | Positive | positive | Negative | 15% | 1+ | None | None | 2+ | 3+ | 2+ | None | None | None | None | None | None | None |  |
| 5 | Positive | Negative | Negative | 20% | 2+ | None | None | 2+ | 2+ | Negative | None | 1+ | None | None | 1+ | None | None |  |
| 6 | Negative | Negative | Negative | 5% | 1+ | None | None | 1+ | None | None | None | None | 1+ | 1+ | None | 1+ | None |  |
| 7 | Negative | Negative | Negative | 10% | 2+ | None | None | 3+ | 2+ | None | None | None | Negative | 3+ | None | Negative | 1+ |  |
| 8 | Positive | Negative | Negative | 10% | 1+ | None | None | 2+ | 2+ | 1+ | None | 1+ | None | 2+ | None | 2+ | None |  |
| 9 | Positive | Negative | Negative | 15% | 1+ | 1+ | None | 3+ | 1+ | None | None | None | None | None | 1+ | None | None |  |
| 10 | Pegative | Negative | Negative | 35% | 2+ | None | None | None | Focal (1+) | Negative | None | None | None | 1+ | None | None | None |  |
| 11 | Positive | Negative | Negative | 5% | 1+ | 3+ | None | None | 3+ | 2+ | None | 1+ | None | None | None | None | None |  |
| 12 | Negative | Negative | Negative | 10% | 3+ | None | None | 2+ | 2+ | 1+ | None | None | None | 3+ | None | None | None |  |
| 13 | Negative | Negative | Negative | 10% | 1+ | None | 2+ | None | None | None | None | 3+ | 3+ | None | None | None | None |  |
| 14 | Negative | Negative | Negative | 10% | 1+ | None | None | None | None | None | None | None | None | None | None | None | None |  |
| ER, estrogen receptor; PR, progesterone receptor; HER2, human epidermal growth factor receptor 2; 1+, negative staining with focally very weak immunoreactivity; 2+, medium positive immunostaining; 3+, strongly positive immunostaining. | | | | | | | | | | | | | | | | | | |
